# Path towards mRNA delivery for cancer immunotherapy from bench to bedside

**DOI:** 10.7150/thno.89247

**Published:** 2024-01-01

**Authors:** Wenfei Chen, Yining Zhu, Jinhan He, Xun Sun

**Affiliations:** 1 Department of Pharmacy, Institute of Metabolic Diseases and Pharmacotherapy, West China Hospital, Sichuan University, Chengdu 610041, China.; 2 Key Laboratory of Drug-Targeting and Drug Delivery System of the Education Ministry, Sichuan Engineering Laboratory for Plant-Sourced Drug and Sichuan Research Center for Drug Precision Industrial Technology, West China School of Pharmacy, Sichuan University, Chengdu 610041, China.; 3 Department of Biomedical Engineering, Johns Hopkins University School of Medicine, Baltimore, MD 21218, USA.

## Abstract

Messenger RNA (mRNA) has emerged as a promising therapeutic agent for the prevention and treatment of various diseases. mRNA vaccines, in particular, offer an alternative approach to conventional vaccines, boasting high potency, rapid development capabilities, cost-effectiveness, and safe administration. However, the clinical application of mRNA vaccines is hindered by the challenges of mRNA instability and inefficient *in vivo* delivery. In recent times, remarkable technological advancements have emerged to address these challenges, utilizing two main approaches: *ex vivo* transfection of dendritic cells (DCs) with mRNA and direct injection of mRNA-based therapeutics, either with or without a carrier. This review offers a comprehensive overview of major non-viral vectors employed for mRNA vaccine delivery. It showcases notable preclinical and clinical studies in the field of cancer immunotherapy and discusses important considerations for advancing these promising vaccine platforms for broader therapeutic applications. Additionally, we provide insights into future possibilities and the remaining challenges in mRNA delivery technology, emphasizing the significance of ongoing research in mRNA-based therapeutics.

## Introduction

Immunotherapy has become an attractive strategy for preventing and treating a wide range of diseases 1. One area where it has shown promise is in the development of cancer vaccines, which harness the body's immune system to recognize and eliminate cancer cells 2, 3. Recent clinical trials focusing on immune checkpoint blockade and adoptive T cell transfer have demonstrated the effectiveness of vaccines targeting tumor-associated or tumor-specific antigens (TAAs or TSAs) 4, 5. These vaccines have the ability to specifically target and destroy malignant cells, leading to therapeutic immune responses. Despite decades of research and development in the field of cancer vaccines, their clinical translation into diverse therapies has proven to be challenging due to the highly variable nature of tumor antigens and the relevantly low immune responses within the tumor microenvironment (TME) 6, 7. Conventional vaccine treatments such as live attenuated and inactivated pathogens or subunit vaccines have shown long-lived protective immunity against tumor antigens 8, 9. However, there are still significant obstacles to overcome in order to apply these approaches to cancer diseases. Therefore, there is an urgent need for advancements in potent and versatile vaccine platforms.

Nucleic acid-based therapeutics have emerged as promising alternatives to conventional vaccine approaches. Messenger RNA (mRNA), discovered through pioneering studies from 1947 to 1961, serves as a transient intermediary between genes and proteins 10. In the late 1980s, extensive research into the structure and function of mRNA led to the development of *in vitro*-transcribed (IVT) mRNA 11. Following the first proof-of-concept study in animals in 1990, multiple strategies have been explored to enhance the stability and reduce the immunogenicity of IVT mRNA 8, 12. Unlike DNA vaccines, mRNA vaccination leads to the transient expression of encoded proteins, thus avoiding complications associated with insertional mutagenesis. Moreover, mRNA vaccines offer the flexibility to specifically design and encode diverse peptide and protein structures, enabling the expression of complete antigens. By presenting a greater number of epitopes through class I and class II major histocompatibility complex (MHC) molecules, mRNA vaccines hold potential to elicit more intensive cellular and humoral responses compared to peptide antigen vaccines 13, 14. mRNA vaccines have predominantly demonstrated safety and good tolerance profiles, with only a few serious concerns. Nevertheless, the clinical experience regarding their acute and long-term side effects remains somewhat limited, which may result in localized or systemic inflammatory reactions in mammalian organisms 12.

In recent years, mRNA vaccination has undergone significant advancements in preclinical and clinical trials 11, 15. However, the successful translation of mRNA cancer therapeutics from the laboratory to the market was not straightforward, which has faced two major challenges. First, there is insufficient intracellular protein expression due to the catalytic hydrolysis of mRNA. Second, there is inadequate antigen loading and maturation of antigen-presenting cells (APCs) for the subsequent immune activation. To address these challenges and improve mRNA delivery as well as *in vivo* protein expression, significant progress has been made in the development of nucleic acid drug delivery systems. These advancements have accelerated the preclinical and clinical applications of mRNA therapeutics, establishing mRNA as a new class of drug 16, 17. A variety of strategies have been developed for the mRNA vaccine delivery with high efficiency and safety, including encapsulation of mRNA in nanoparticles, in viral, bacterial or cell-mediated vectors, and sequence optimization for increased stability and tailored immunogenicity 18-20. Additionally, materials science has played a crucial role in designing mRNA delivery vectors, utilizing lipids, lipid-like materials, polymers, and protein or peptide derivatives 3, 21, 22. Lipid nanoparticles (LNPs) have been extensively investigated and applied in clinical settings for delivering small molecules, small interfering RNA (siRNA) drugs, and mRNA vaccines 23-25. With the advancement of scale-up manufacturing, mRNA vaccines offer several advantages over other vaccine techniques, including rapid, cost-effective production as well as the potential for large-scale deployment. Currently, non-replicating mRNAs have been predominantly investigated in clinical trials for cancer treatments, while self-amplifying mRNAs (SAMs) have also gained extensive attention due to their long-lasting efficacy and potential for reduced dosages 8, 26.

The mRNA vaccine field has entered a phase of rapid development, accumulating a substantial body of preclinical studies and initiating multiple human clinical trials 27, 28. Technological and pharmaceutical engineering innovations have made the mRNA vaccine as a feasible candidate. In this review, we will discuss the basic pharmacology of mRNA vaccines and recent advances in mRNA vaccine technology. We will also summarize the delivery systems of mRNA cancer vaccines and highlight important examples of mRNA cancer vaccines in preclinical and clinical studies. Lastly, we will provide considerations and challenges for clinical translation, and further present perspectives on the future of mRNA vaccines.

## Fundamental pharmacology of mRNA vaccine

**Figure 1:**
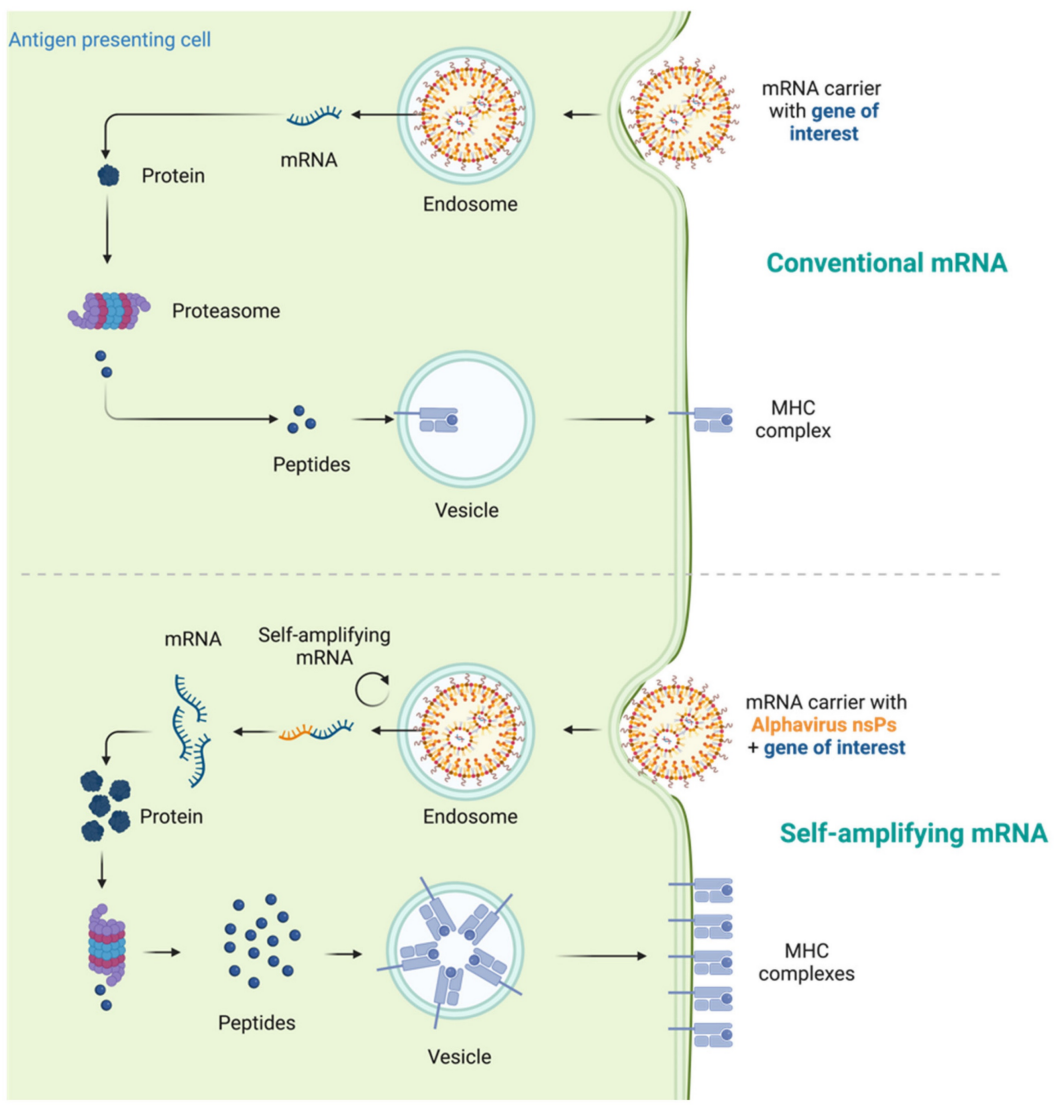
** Mechanism comparison of conventional mRNA vaccines and self-amplifying mRNA (SAM) vaccines.** Traditional mRNA-based vaccines typically encode the antigen of interest along with 5ʹ and 3ʹ untranslated regions (UTRs). In contrast, SAMs encompass not only the antigen but also the viral replication machinery, facilitating intracellular RNA amplification and enhancing protein production. After internalization and arrival in the cytosol, the mRNA is recognized by ribosomes, leading to its translation into proteins, which then undergo post-translational modifications to attain the correct folding and functionality.

mRNAs serve as the intermediate step between the translation of protein-encoding DNA and the production of proteins by ribosomes in the cytoplasm. For cancer vaccines, three types of mRNAs have been extensively studied: non-replicating unmodified mRNA, modified mRNA, and virus derived self-amplifying mRNA (SAM) 8. Conventional mRNA-based vaccines encode the antigen of interest and contain 5ʹ and 3ʹ untranslated regions (UTRs), while SAMs encode both the antigen and the viral replication machinery, enabling intracellular RNA amplification and increased protein expression (Figure 1) 29, 30.

*In vitro* transcription (IVT) technology is commonly used to synthesize both non-replicating mRNA (modified or unmodified) and SAM 4, 31. Briefly, IVT mRNA is produced from a linear DNA template using a T7, a T3 or an Sp6 phage RNA polymerase 8, 32. IVT production simplifies and expedites mRNA production compared to large-scale protein production and purification, as it does not require cells and associated regulatory hurdles 33, 34. Non-replicating IVT mRNA typically consists of an open reading frame (ORF) region that encodes the target antigen sequences 35, flanked by five-prime (5′) and three-prime (3′) untranslated region (UTR) 36, and further stabilized by 7-methylgaunosine (m7G) 5′ cap and 3′ poly (A) tails respectively 37, 38. In contrast, SAM contains two ORFs, one encoding the targeted antigen sequences and the other encoding viral replication machinery, enabling long-lasting RNA amplification intracellularly. Once the mRNA is internalized and reaches the cytosol, it will be recognized by ribosomes and subsequently translated into proteins, which undergo post-translational modifications to become properly folded and functional 39. This unique pharmacological feature of mRNA is beneficial for cancer vaccines, as it facilitates the delivery of cytosolic or transmembrane proteins to the correct cellular compartments, ensuring proper presentation and functionality. The remaining IVT mRNA template can be degraded through normal physiological processes, minimizing the risk of metabolite toxicity.

However, there are several limitations to consider when using mRNA for vaccine improvements 40, 41. First, naked mRNA is susceptible to degradation by extracellular RNases, and is not efficiently internalized by APCs. This hampers the effectiveness of mRNA vaccines in generating an immune response. Second, mRNA possesses intrinsic immunogenicity, leading to the activation of the interferon (IFN)-related pathway and triggering innate immunity. While this immunogenicity can be beneficial in promoting immune activation, it may also result in the premature degradation of mRNA and reduced antigen expression. In addition, the impurities, mainly produced by the double stranded RNA (dsRNA) during IVT process, are likely to activating the innate immunity and impeding mRNA translation. In the following sections, we will discuss functional strategies to overcome these limitations.

## Major improvements in mRNA vaccine technology

Efficient *in vivo* delivery of mRNA is crucial for achieving desired therapeutic responses 42, 43. The challenges associated with mRNA vaccine delivery are primarily centered around the need to protect the mRNA from degradation by ubiquitous endonucleases, ensure its successful delivery to target cells, facilitate endocytosis, and enable escape from endosomes to avoid premature degradation. Moreover, the uptake mechanism of mRNA varies depending on the cell type, and the physicochemical properties of mRNA complexes can significantly influence their cellular delivery and distribution within organs 44. Currently, two main approaches are employed for mRNA vaccine delivery: the *ex vivo* loading of mRNA into dendritic cells (DCs) followed by re-infusion of transfected cells, and the direct injection of mRNA, either with or without a carrier 45, 46.

The *ex vivo* loading of mRNA into DCs offers several advantages, including precise control over the cellular target, transfection efficiency, and other cellular conditions 47-49. However, this approach is relatively expensive and labor-intensive, making it less suitable for large-scale vaccination. On the other hand, the direct injection of mRNA is a more rapid and cost-effective method, but it currently lacks the ability to achieve precise and efficient cell-type specific delivery 50-52. To overcome these challenges, extensive research has focused on validating mRNA vaccine platforms in recent years. Significant progress has been made in developing highly efficient and non-toxic RNA carriers that enable prolonged antigen expression *in vivo*. Some vaccine formulations also incorporate novel adjuvants, while others demonstrate potent immune responses even in the absence of known adjuvants 53. The following section provides a summary of the key advancements in mRNA loading and delivery technologies and their impact on vaccine efficacy.

### *Ex vivo* transfection of mRNA on DCs

**Figure 2:**
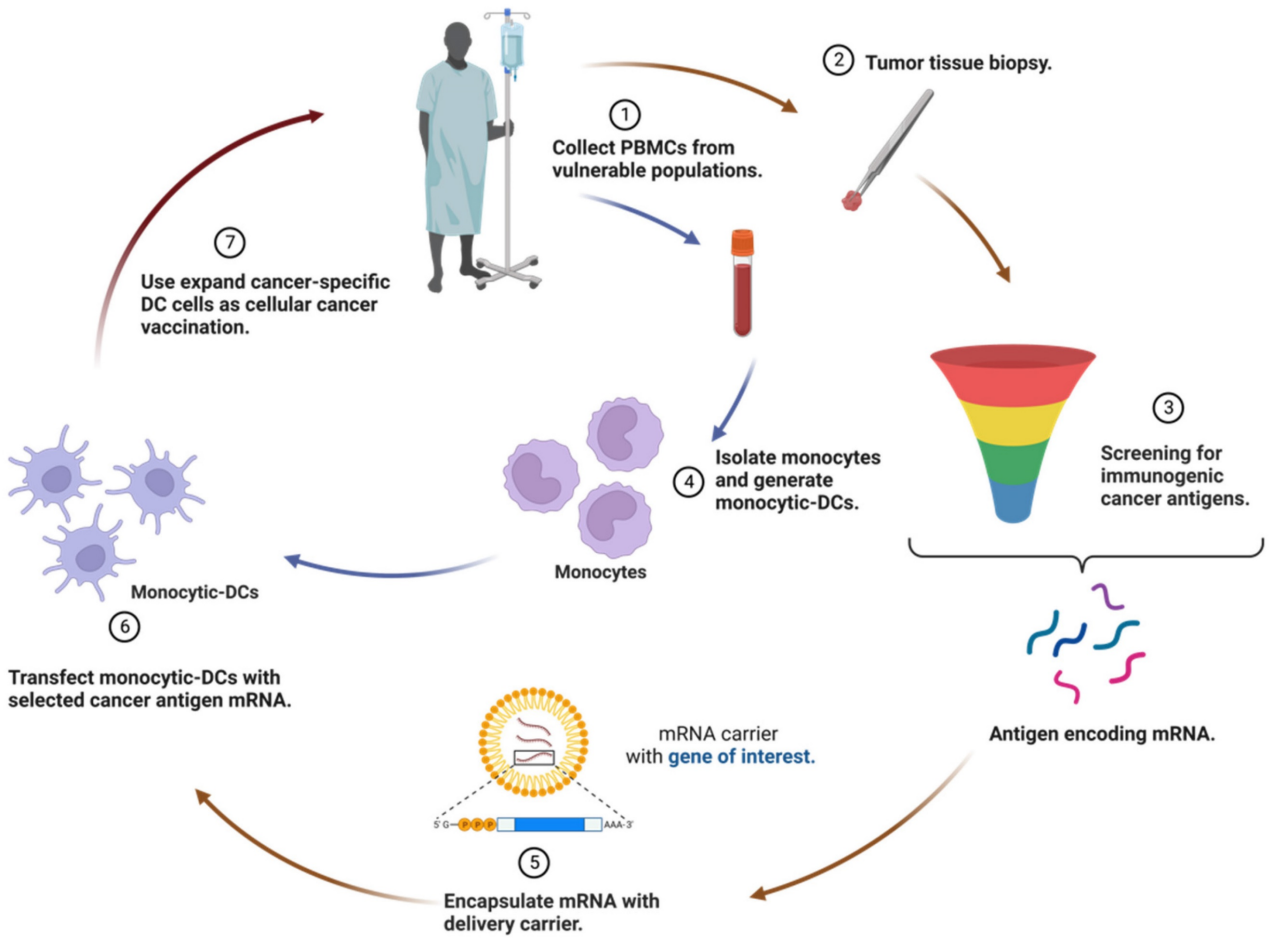
** Therapeutic regimen of *ex vivo* transfection of mRNA on dendritic cells (DCs).** The utilization of DCs as a vaccination platform entails the transfection of DCs with mRNA encoding tumor antigens, followed by their delivery to the host, thereby triggering an immune response against the specific antigen. After successful *ex vivo* transfection of DCs with mRNA, these modified cells are re-infused into the recipient as an autologous vaccine, with the aim of eliciting immune responses. Consequently, most *ex vivo*-transfected DC vaccines predominantly foster a cell-mediated immune response, a valuable feature for the treatment of cancer.

DCs play a crucial role as potent APCs in the immune system. Leveraging DCs as a vaccination platform involves transfecting them with mRNA encoding tumor antigens and delivering them to the host, stimulating an immune response against the specific antigen 45, 54. While DCs are able to internalize naked mRNA via different endocytic pathways, *ex vivo* transfection commonly utilizes electroporation to achieve high transfection efficiency without a carrier. Once DCs are successfully transfected with mRNA *ex vivo*, they are re-infused into the recipient as part of an autologous vaccine to induce immune responses (Figure 2). Notably, most *ex vivo* transfected DC vaccines predominantly promote a cell-mediated immune response, which is beneficial for treating cancer diseases 55.

As DCs are conducive to initiating antigen-specific immune responses, it seems logical to utilize them for cancer immunotherapy. The pioneering study in 1996 demonstrated that DCs electroporated with mRNA elicited a potent immune response against the tumor antigen 56. In this study, mice vaccinated with DC pulsed with RNA from ovalbumin (OVA)-expressing tumor cells were protected against a challenge with OVA-expressing tumor cells, demonstrating the potential of RNA-pulsed DC-based vaccines for patients with small, possibly microscopic, tumors. Besides, mRNA-encoded adjuvants, which effectively enhance the potency of DC cancer vaccines, have been identified. Several studies verified that the electroporation of DCs with mRNAs encoding costimulatory molecules such as CD83 57, tumor necrosis factor receptor superfamily member 4 (TNFRSF4; also known as OX40)^58^, and 4-1BB ligand (4-1BBL)^59^ led to a substantial increase in the immune stimulatory activity of DCs. Then DCs were functionalized by incorporating mRNA-encoded pro-inflammatory cytokines, such as interleukin (IL)-12, into the vaccination strategy 60, 61. Moreover, a notable example is the use of TriMix, which is a combination of mRNA-encoded adjuvants including CD70, CD40L, and constitutively active toll-like receptor 4 (caTLR4). TriMix can be electroporated together with antigen-encoding mRNA or multiple mRNAs, promoting the immune response generated by DCs and enhancing their antigen-presenting capabilities 62, 63. This formulation proved that these DCs were effective in inducing naive CD4+ T cells to differentiate into IFN-γ-secreting type 1 T helper (Th1) cells. In a phase IB clinical trial, patients with pretreated advanced melanoma tolerated administration of DCs transfected with mRNA encoding melanoma-associated antigens and TriMix adjuvant (referred to as TriMixDC-MEL) well 64. Encouragingly, the outcome has advanced to phase II clinical trials to further assess the safety and activity of adjuvant TriMixDC-MEL in advanced melanoma patients 65, 66. The success of these strategies underscores the importance of exploring optimized autologous monocyte-derived DC formulations in combination with approved adjuvant therapies.

### Delivery systems of mRNA cancer vaccine

Compared to the approach of *ex vivo* loading mRNA into DCs, the direct injection of mRNA offers advantages in terms of speed and cost-effectiveness. Naked mRNA-based systems have been successfully used for *in vivo* immunizations, particularly in formats that preferentially target APCs via intradermal or intranodal injections, but one limitation of them can be the short extracellular half-life of naked mRNAs 67, 68. To overcome this limitation and enhance the delivery efficiency of mRNA cancer vaccines, researchers have developed a range of viral and non-viral vehicles 69, 70. Viral vector-based technologies have shown promise in delivering nucleic acid vaccines into cells 71. However, their application is often restricted by pre-existing or vaccine-induced anti-vector immunity, which may diminish vaccine efficacy. Synthetic delivery vehicles such as liposomes, lipid nanoparticles, polymers, and other newly produced vesicles have been developed for the delivery of mRNAs to address those challenges 72-74. In the subsequent section, we will delve into the preclinical applications of non-viral vectors for mRNA vaccine delivery.

#### Liposome-based delivery

Liposomes and their derivatives are widely used as vectors for mRNA cancer vaccines, and their design and optimization remain an active area of research to improve antigen expression *in vivo* 75. For example, the constructed liposome-mRNA encoding human carcinoembryonic antigen (CEA) complexes first confirmed the proof-of-concept of mRNA cancer vaccines in preclinical studies 76. This strategy is particularly promising for eliciting an immune response against proto-oncogene products or growth factors that may pose a risk of inducing malignant transformation due to prolonged protein expression. By utilizing mRNA-based vaccines, it becomes possible to precisely control and limit the duration of protein expression, thereby reducing the potential risks associated with prolonged exposure to proto-oncogene products or growth factors. One strategy involves the use of mannosylated and histidylated lipopolyplexes (Man(11)-LPR100), which were obtained from adding mannosylated and histidylated liposomes to mRNA-PEGylated histidylated polylysine polyplexes 77. When injected intravenously, mRNA-loaded Man(11)-LPR100 demonstrated higher transfection efficiency in DCs and exhibited better antitumor effects in animal models compared to sugar-free LPR100. Lipid-Polymer-RNA lipopolyplexes (LPR) are attractive for mRNA delivery systems, and incorporating mannose-containing glycolipids, which are specific to endocytic receptors present on the surface of DCs, is a valuable strategy. In 2018, trimannosylated-LPR was shown to induce more effective transfection with antigens, to recruit more DCs into the draining lymph nodes, and to promote stronger antigen-specific immune responses compared to monomannosylated-LPR 78. In another work, mannose cholesterol conjugates (MPn-CHs) were used to prepare DC-targeted liposomes (MPn-LPs) as mRNA carriers 79. The results indicated that MP1000-LPX enhanced mRNA expression primarily through the over-expressing mannose receptor (CD206) on the surface of DCs, suggesting its potential as a DC-targeting delivery system for mRNA vaccine after rational design. In a recent preclinical study, DOTAP/DP7-C liposomes were employed as both the carrier and the adjuvant, loaded with mRNA encoding five tumor neoantigens of the mouse LLC cell line LL2 (DOTAP/DP7-C/LL2) 80. This formulation efficiently delivered mRNA to different type of DCs, stimulated DC maturation, and promoted the secretion of pro-inflammatory cytokines. Subcutaneous administration of DOTAP/DP7-C/LL2 neoantigen encoding mRNA complexes significantly inhibited the growth of LL2 tumors and also activated the antigen-specific lymphocyte reactions, which were superior to those of DOTAP/LL2 neoantigen-encoding mRNA complex.

#### Lipid nanoparticle-based delivery

**Figure 3:**
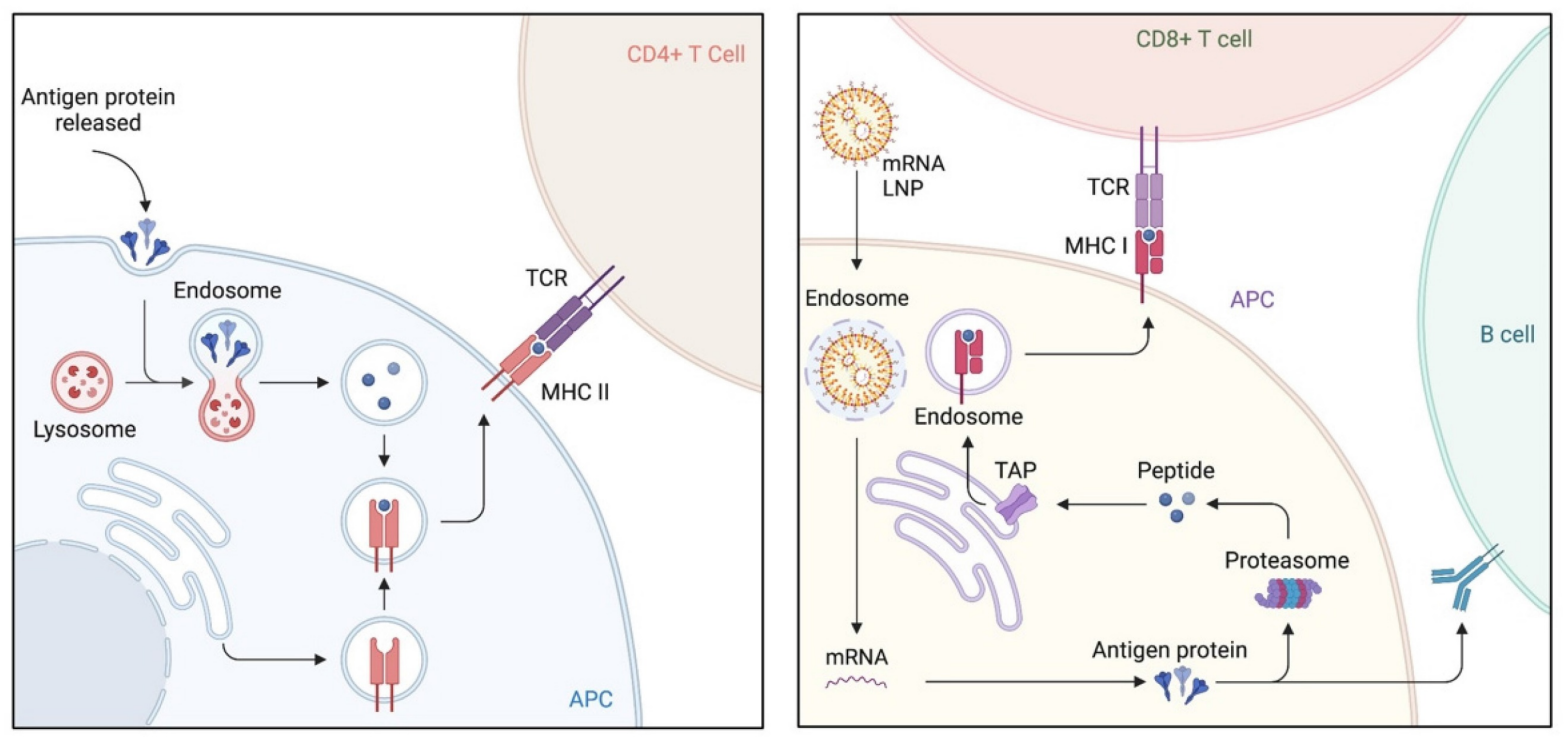
** Immunologic mechanism of lipid nanoparticles (LNPs), emerged as the most promising and widely used mRNA delivery platforms.** LNPs excel in the efficient delivery of mRNA by merging with the lipid bilayer of early endosomes, thereby facilitating the transport of mRNA into the cytosol. Upon vaccination and uptake by antigen-presenting cells (APCs), the mRNA is transported to the cytoplasm, where it undergoes antigen processing and enters the major histocompatibility complex (MHC) presentation pathway. This results in the presentation of antigens by APCs on MHC class I and II molecules, subsequently activating CD8+ T and CD4+ T cells, respectively. CD4+ T cells also play a role in stimulating antigen-specific B cells, leading to the initiation of a humoral immune response.

Lipid nanoparticles (LNPs) have emerged as the most promising and widely used mRNA delivery platforms due to the successful applications of mRNA-LNP vaccines against SARS-CoV-2 81, 82. LNP carriers are approximately 100 nm in size and primarily consist of four components: ionizable lipids, lipid-linked polyethylene glycol (PEG), cholesterol, and phospholipids 83. LNPs efficiently deliver mRNA by fusing with the lipid bilayer of early endosomes, allowing for the transport of mRNA into the cytosol (Figure 3). This intracellular delivery enables the translation of mRNA into functional proteins within the target cells 84, 85. Ongoing research and development in this field are expected to drive further advancements in the delivery of therapeutic RNA molecules.

The success and potency of LNPs for mRNA delivery are attributed to their key components. First, ionizable lipids are the most important component of LNPs as they determine their potency and differentiate mRNA-LNPs 23, 86. They consist of hydrophilic head groups and hydrocarbon chains to enhance self-assembly, as well as linkers to connect them. In LNPs, ionizable lipids remain unionized and complexed with mRNA to form stable lipoplexes. During systemic circulation at neutral pH (~7.4), ionizable lipids used in mRNA-LNP formulations remain in a neutral 19, 72. However, when exposed to the acidic pH environment of early endosomes (pH ~6.5), these lipids undergo protonation, which enables the lipids to facilitate fusion with the endosomal membrane, ultimately leading to the release of the mRNA payload into the cytosol. This pH-dependent activation mechanism allows for efficient intracellular delivery of mRNA, ensuring its successful translation into protein within the target cells. Some ionizable lipids are known to induce inflammation and cell toxicity by activating toll-like receptors (TLR) pathways. Second, PEG lipids generally comprising < 2.5% of the total LNP formulation consist of a hydrophilic PEG-polymer, which is conjugated with a hydrophobic lipid anchor. PEG lipids play an important role in balancing circulation time and cellular uptake, and help to inhibit particle aggregation and improve storage stability. Balancing the PEG lipids should be critical as high concentrations can hinder the transport of RNA into cells. Finally, phospholipids and cholesterol are components that contribute to the structural integrity and phase transition behavior of LNPs. These components also are of great importance in eliciting significant innate immune responses as they are naturally present on mammalian cell membranes.

The modularity and versatility of mRNA-LNP vaccines offer several advantages for their development and application. The components of LNP formulations, including their ratios, targeting moieties, and overall lipid-to-mRNA ratios, can be customized and optimized to suit various targets and applications 87-89. This flexibility allows for the development of tailored LNPs that are specifically designed to enhance vaccine effectiveness. Moreover, LNPs with reduced immunogenicity have the capacity to deliver larger cargoes, enabling the delivery of more complex mRNA constructs. This capability opens up possibilities for the inclusion of multiple antigens or additional therapeutic elements within a single vaccine formulation. Another significant benefit is the potential for rapid and large-scale manufacturing of mRNA-LNP vaccines. The LNP technology offers scalability, which is crucial for meeting the demands of widespread vaccination campaigns, especially during times of public health emergencies.

**Figure 4:**
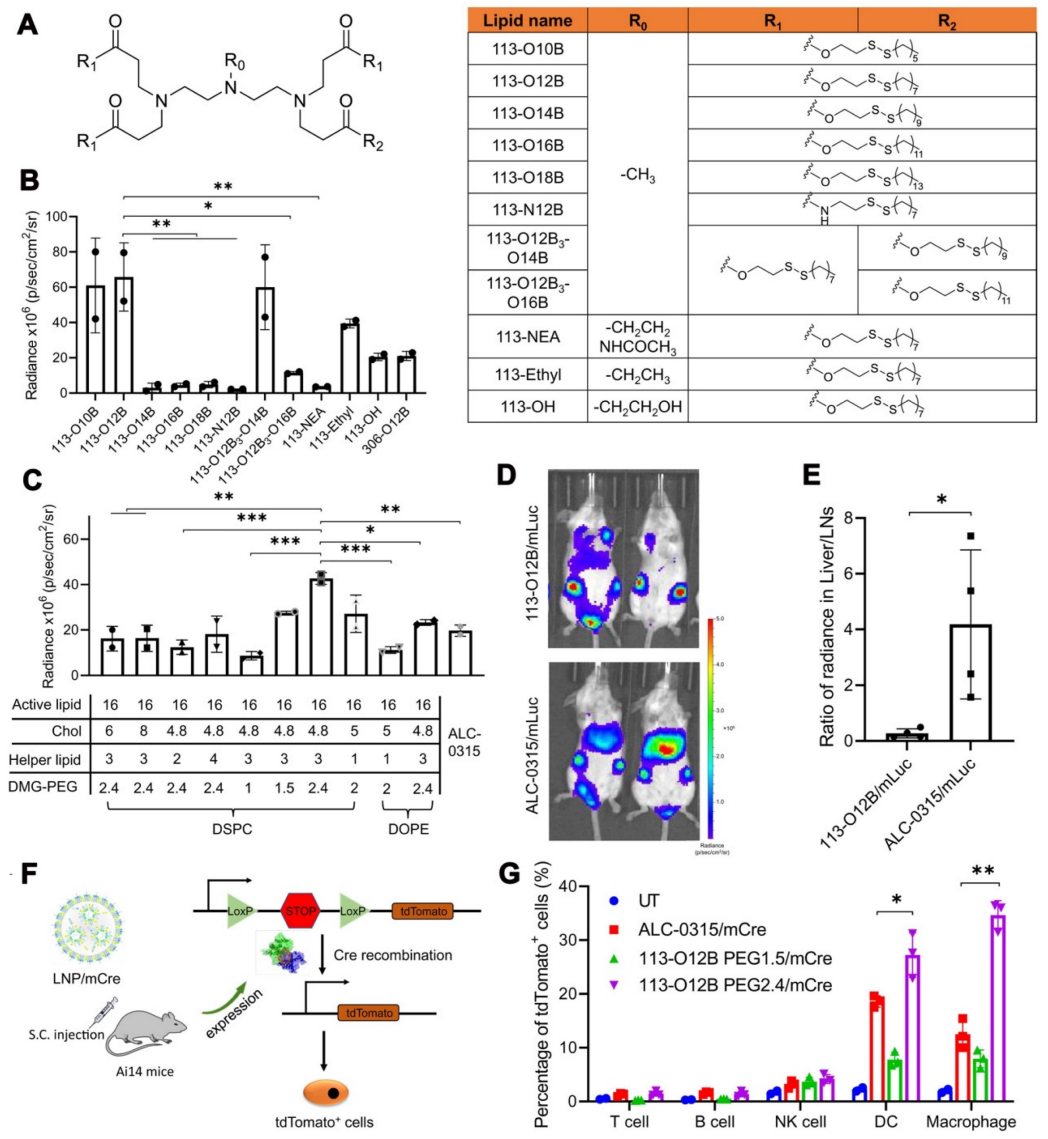
** Optimization of LNPs with lymph node (LN)-targeting specificity for a therapeutic mRNA cancer vaccine.** (A) The chemical structure of lipids used in this study. (B) The bioluminescence within inguinal LNs after treatment with LNP/mLuc subcutaneously at the tail base for 6 h. (C) The bioluminescence within inguinal LNs after treatment by LNP/mLuc with different formulations for 6 h. (D) Representative images of bioluminescence distribution in mice treated with 113-O12B/mLuc and ALC-0315/mLuc for 6 h. (E) Ratio of radiance in liver and inguinal LNs after SC injection of mLuc for 6 h. (F) Mechanism of subcellular analysis of mRNA expression in Ai14 reporter mice. (G) Percentage of tdTomato-positive cells in different types of immunocytes after treatment with LNP/mCre subcutaneously at tail base for 48 h. Adapted with permission from 94. Copyright 2022, National Academy of Science.

Several studies have demonstrated the effectiveness of mRNA-LNP vaccines in preclinical models. In 2017, a lipid nanoparticle formulation for the delivery of mRNA vaccines was constructed and optimized to induce a cytotoxic T cell response 90. The results revealed that LNPs containing mRNAs encoding tumor antigens gp100 and TRP2 combined with LPS as the adjuvant could effectively cause tumor shrinkage and extend the overall survival of vaccinated mice. Additionally, the development of combinatorial libraries of ionizable lipid-like materials led to the identification of mRNA delivery vehicles that enhanced antitumor efficacy and induced APC maturation via the intracellular stimulator of interferon genes (STING) pathway 91. This strategy contributed to the diversity of synthesized lipid structures, and identified the head group as a key component; changing the chemical structure of the lipid head group could tune the immunostimulatory effect of these lipids. Notably, SM-102 and ALC-0315 are the crucial ionizable delivery components in the Moderna mRNA-1273 and Pfizer/BioNTech BNT162b2 vaccines for preventing coronavirus disease 2019 (COVID-19), respectively 23, 92, 93. In a present study, LNP 113-O12B, with lymph node (LN)-targeting specificity, was explored and applied for a therapeutic mRNA cancer vaccine (Figure 4) 94. Compared with LNPs formulated with ALC-0315, 113-O12B demonstrated significantly reduced mRNA expression in the liver and higher expression in LNs after subcutaneous injection. As a result, the targeted delivery of full-length ovalbumin (OVA)-encoding mRNA vaccine remarkably promoted a CD8+ T cell response and showed therapeutic effects against OVA-transduced B16F10 tumor model.

#### Polymer-based delivery

**Figure 5:**
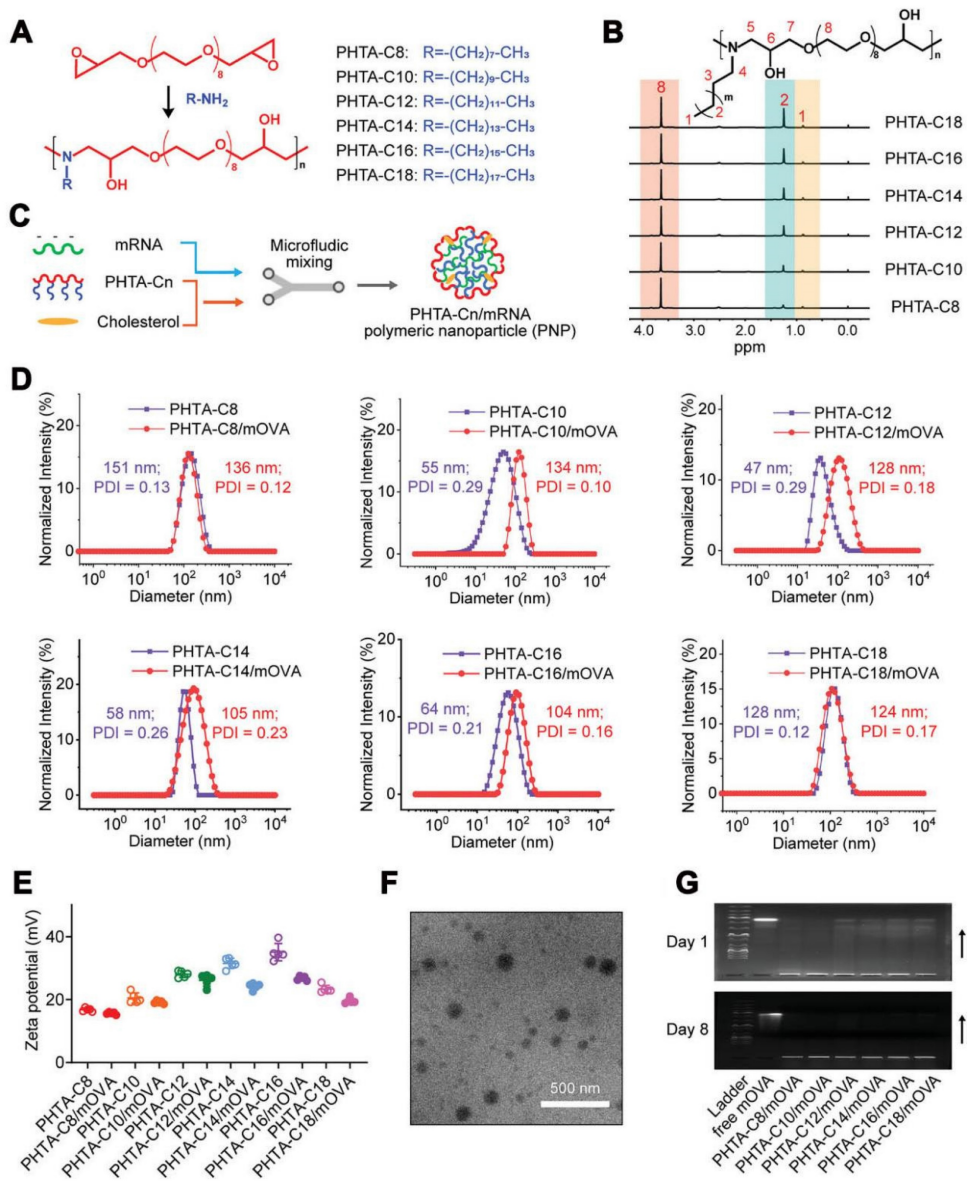
** A series of alternating copolymers “PHTA” featured with ortho-hydroxy tertiary amine (HTA) repeating units for mRNA vaccine delivery in cancer therapy.** (A-C) Design of the PHTA polymers for mRNA vaccine delivery. (D) Size distribution and (E) zeta potential of PHTA-Cn/mOVA before and after OVA mRNA encapsulation. (F) Representative TEM image of PHTA-based polymeric nanoparticle (PNP). (G) Agarose gel electrophoresis images of PHTA-Cn/mOVA on day 1 and day 8 post OVA mRNA encapsulation. Adapted with permission from 103. Copyright 2023, Wiley-VCH GmbH.

Polymeric nanoparticles have been treated as another promising approach for mRNA delivery 95. Early studies focused on cationic polymers such as polyethylenimine or poly-l-lysine, but their toxicity limited their application in mRNA delivery 96. To address this issue, biodegradable poly(β-amino esters) (PBAEs) have been synthesized and used for the *in vivo* delivery of functional mRNAs to circulating T cells and various tissues. For example, PBAE-based nanoparticles showed effectiveness in mRNA delivery and demonstrated potential for therapeutic applications 97, 98. Moreover, the charge-altering releasable transporter (CART) has been investigated as a type of potential polymer for mRNA delivery 99, 100. CARTs are amphiphilic polymers that can be used to generate combinatorial libraries of oligonucleotide transporters with varied lipid domains. These CART-based formulations exhibited prominent transfection efficiency in primary T cells and *in vivo* settings 101, enabling the enhancement of mRNA delivery to lymphocytes. Furthermore, mRNA-CART vaccines encoding whole proteins demonstrated the superior activation of antigen-specific T cells compared to conventional synthetic viral peptide mixtures 102. In the pursuit of potent yet low-inflammatory mRNA cancer vaccine vectors, alternating copolymers known as "PHTA" featured with ortho-hydroxy tertiary amine (HTA) repeating units have been developed (Figure 5) 103. These copolymers aimed to improve the stability of polymeric nanoparticles (PNPs) and prolong their circulation time. Unlike LNPs that can induce inflammation, PHTA-based PNPs demonstrated negligible inflammatory side effects and induced robust CD8+ T cell-mediated antitumor immunity. These PHTA-based PNPs hold promise for the development of mRNA cancer vaccines with improved safety profiles. Notably, polyplex micelles (PMs) were developed by combining ω-cholesteryl (ω-Chol)-poly (ethyleneglycol) (PEG)-polycation block copolymers with mRNA prehybridized with cholesterol (Chol)-tethered RNA oligonucleotides (Chol (+)-OligoRNA) to improve the tolerance and stability of mRNA 104. These PMs showed efficient mRNA introduction into the lungs via intratracheal administration, demonstrating their potential for *in vivo* applications.

#### Protamine-based delivery

Protamine, a cationic peptide, has been utilized to protect mRNA from degradation by serum RNases; however, protamine-complexed mRNA alone revealed unsatisfied protein expression and antitumor efficacy in animal models, possibly due to an excessively strong association between protamine and mRNA, which may result in limited dissociation and hinder efficient mRNA release 15, 105. Practically, protamine can be combined with liposomes to enhance mRNA delivery and expression. For instance, the liposome-encapsulated condensed RNA-peptide complex led to protein expression in local tissues, and induced antigen-specific cellular and humoral immune responses on animal bodies 106. This approach indicated that both naked and protected RNA could elicit a specific immune response *in vivo*, with the protected RNA remaining stable for longer periods *in vitro*. Another strategy involves the use of cationic liposome/protamine complex (LPC) that exhibit high uptake efficiency of vaccine particles *in vitro* and enhance DC maturation 107. Intranasal immunization of mice with cationic LPC containing mRNA encoding cytokeratin 19 provoked a strong cellular immune response and inhibited tumor growth in an aggressive lung cancer model. This finding provides a foundation for cancer vaccination in humans. Furthermore, a novel carrier called virus-like vaccine particle (VLVP) was designed to resemble a cancer-fighting virus both in structure and activity 108. The VLVPs consisting of antigen-encoding mRNA molecules complexed with positively charged protamine, were then coated with a lipid bilayer formed by both ionizable and non-charged phospholipids. This carrier demonstrated significant efficacy in treating murine tumor models and triggered robust activation of CD8+ T cells.

#### More systems for mRNA delivery

**Figure 6:**
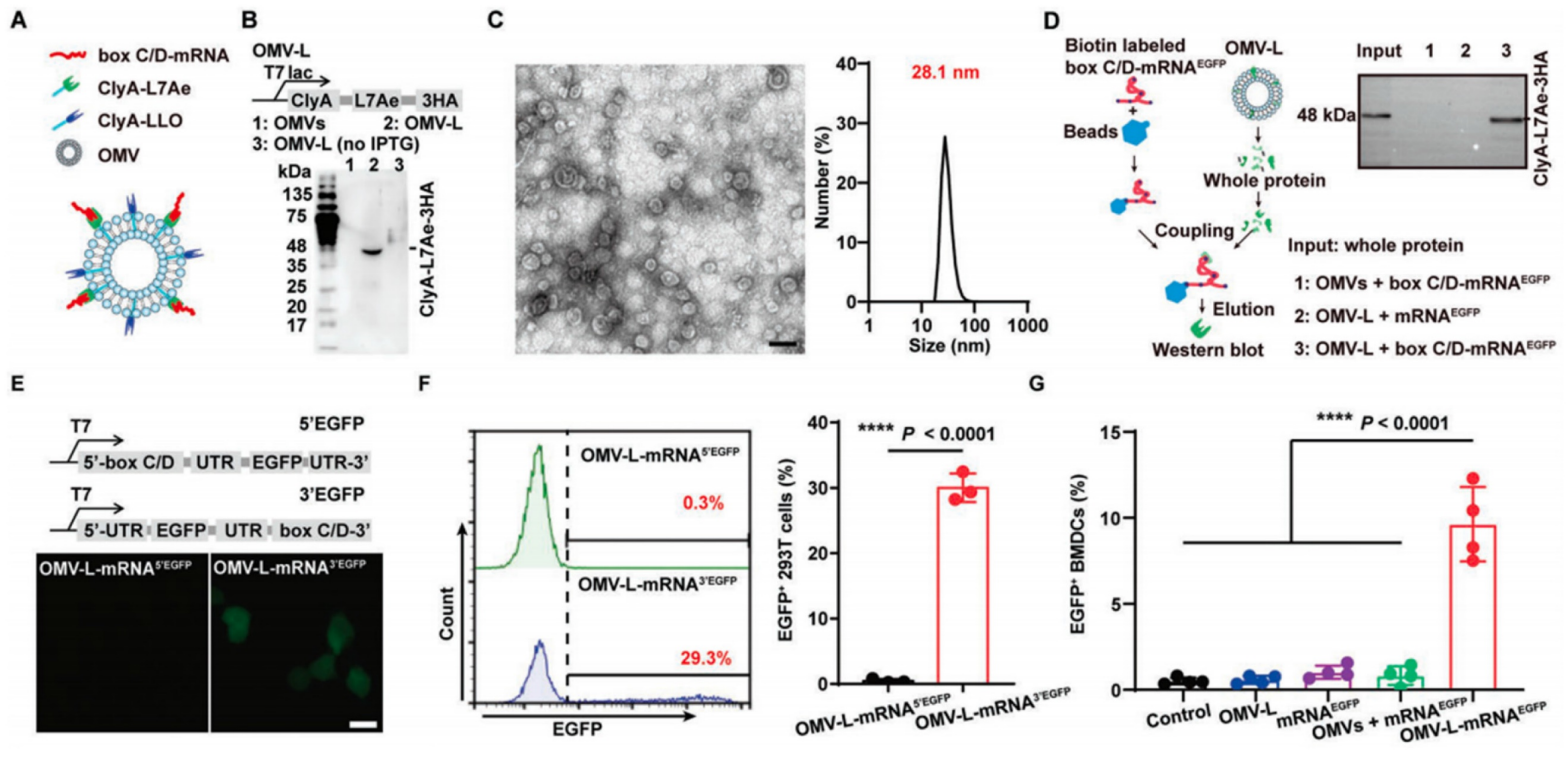
** Design of bacteria-derived outer membrane vesicles (OMVs) as a platform for mRNA delivery.** (A) Schematic illustration of the OMV-based mRNA delivery system. (B) Western blot analysis of ClyA-L7Ae-3HA expression in the L7Ae-modified OMVs (OMV-L) using an anti-HA antibody. (C) Representative TEM image of OMV-L and its size distribution. Scale bar: 50 nm. (D) RNA pull down assay evaluating the binding of OMV-L and box C/D-mRNAEGFP using an anti-HA antibody. (E) Confocal microscopy images and (F) flow cytometry analysis of 293T cells incubated with OMV-L-mRNA3′EGFP or OMV-L-mRNA5′EGFP for 24 h. Scale bar: 10 µm. (G) Expression of EGFP in BMDCs incubated with the indicated formulations 24 h, analyzed by flow cytometry. Adapted with permission from 117. Copyright 2022, Wiley-VCH GmbH.

Many types of vectors have been developed to efficiently and safely deliver mRNA, including extracellular vesicles (EVs), cell membrane vesicles, outer membrane vesicles (OMVs), calcium phosphate (CaP), silica nanoparticles, and other particle-based systems. Among them, EVs have gained considerable attention in the field of mRNA delivery due to their unique biology and role in cell-cell communication. EVs have the capability to carry a variety of cargos, including RNA, DNA, proteins, and lipids, which can be taken up by recipient cells 109-111. For instance, EVs engineered with a high-affinity anti-HER2 scFv antibody (ML39) were applied to deliver HchrR6 mRNA to recipient cells and tumors, showcasing their potential as targeted mRNA delivery vehicles 112. Exosomes, a type of nanoscale EVs, have been extensively studied as carriers for drug delivery 113. For example, exosomes released by reactive astrocyte (RAS) were used to deliver O6-methylguanine DNA methyltransferase (MGMT) mRNA to MGMT-negative glioma cells, effectively overcoming the temozolomide resistance 114. Then a cellular-nanoporation method was reported for the production of large quantities of exosomes containing therapeutic mRNAs and targeting peptides 115. In orthotopic phosphatase and tensin homologue (PTEN)-deficient glioma mouse models, these mRNA-containing exosomes restored tumor-suppressor function, enhanced inhibition of tumor growth, and prolonged survival, underscoring their potential for mRNA-based therapies. Biological membrane coating has emerged as a promising top-down approach for nanocarriers with enhanced biointerfacing capabilities. By coating nanoparticles with cell membranes, researchers created cell membrane-coated nanoparticles capable of displaying viral components such as hemagglutinin (HA), enabling these nanocarriers to mimic viruses and exhibit properties such as virus-mimicking endosomal escape and enhanced cytosolic delivery 116. In a recent study, researchers utilized bacteria-derived outer membrane vesicles (OMVs) as a platform for mRNA delivery (Figure 6) 117. They genetically engineered the OMVs to express specific surface proteins, including the RNA binding protein L7Ae and the lysosomal escape protein listeriolysin O (OMV-LL). Such modification allowed the OMVs to successfully bind and protect the mRNA payload, as well as facilitate its escape from the lysosomal compartment. This innovative bacterial-mediated delivery technology presents a distinct alternative to LNPs for personalized mRNA tumor vaccination. The "Plug-and-Display" strategy employed by OMVs enables their versatile application in mRNA-based vaccines.

**Figure 7:**
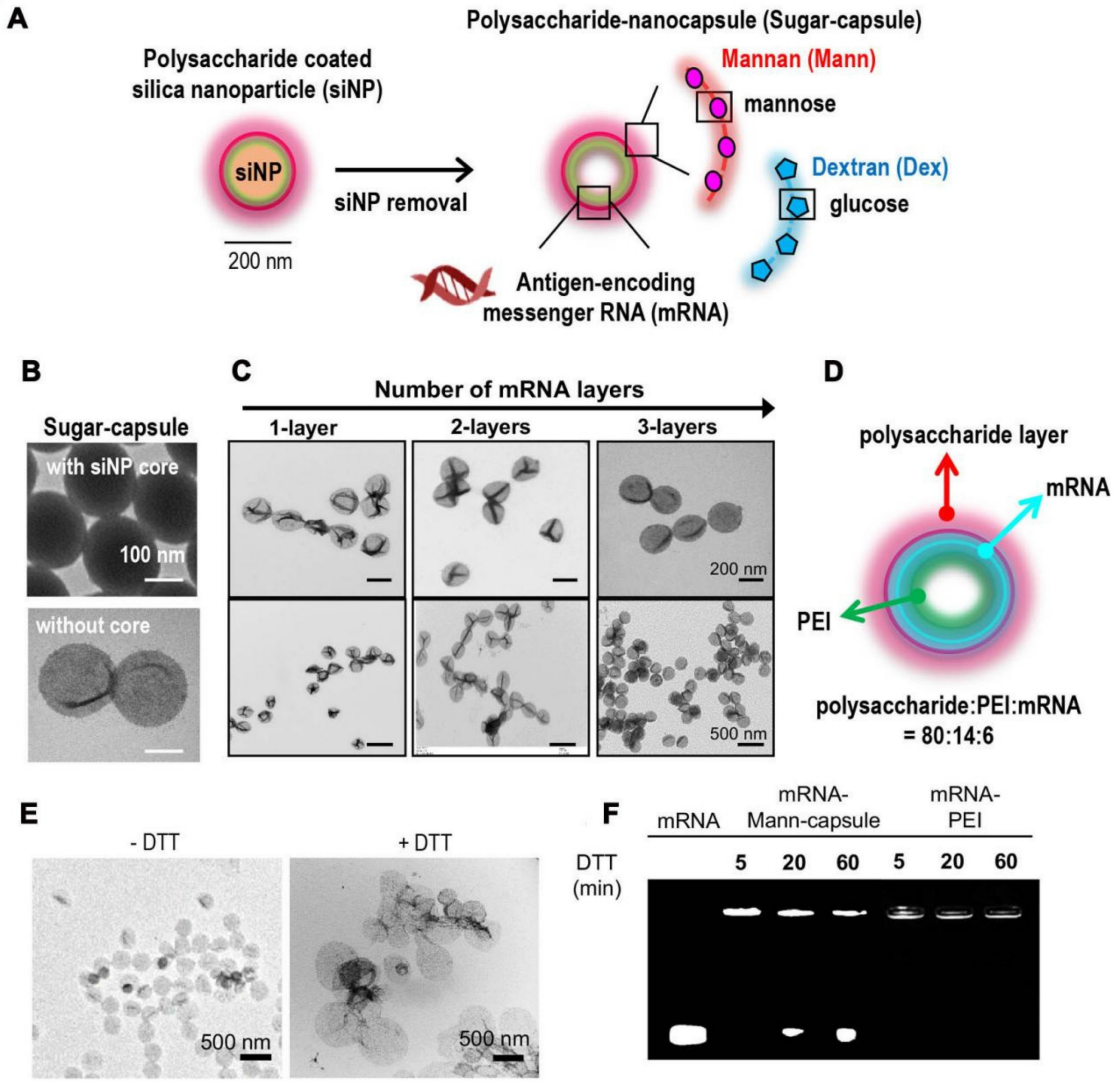
** Synthetic sugar-capsules composed of a mannan or dextran polysaccharide shell with a hollow core serving as a novel nanosystem for mRNA vaccine delivery.** (A) Schematic illustration of synthesis of mRNA-loaded sugar-capsules. (B) TEM images of sugar-capsules before (top) and after (bottom) removal of a core silica nanoparticle. (C) TEM images of sugar-capsules with multilayered mRNA loading at high (top) and low (bottom) magnifification. (D) Illustration of an mRNA-sugar-capsules with the weight ratio of components. (E) TEM images of Mann-capsules and (F) agarose gel image of mRNA-Mann-capsules or mRNA-PEI after DTT treatment. Adapted with permission from 122. Copyright 2020, American Chemical Society.

**Figure 8:**
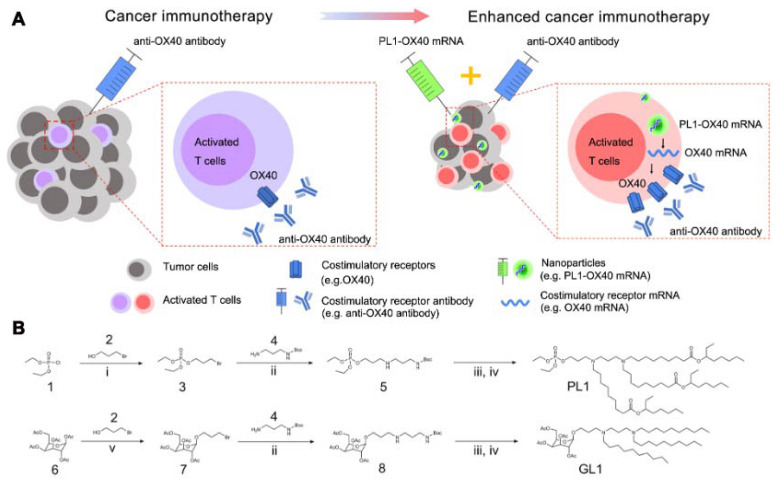
** A library of biomimetic nanoparticles which delivered mRNA encoding costimulatory receptors and enhanced T cell mediated cancer immunotherapy.** (A) Illustration of enhanced cancer immunotherapy via nanoparticles delivering costimulatory receptor mRNA followed by the injection of agonistic antibodies to costimulatory receptors (e.g., PL1-OX40 mRNA + anti-OX40 antibody). (B) Representative synthetic routes to biomimetic compounds: phospholipid and glycolipid derivatives. i. Et_3_N, Toluene, RT. ii. Et_3_N, DMF, RT. iii. TFA, CH_2_Cl_2_, RT. iv. aldehyde, Et_3_N, THF, NaBH(OAc)_3_. Adapted with permission from 123. Copyright 2021, Springer Nature.

In addition, lipid-coated calcium phosphate (LCP) nanoparticles (NPs) were prepared as carriers to deliver mRNA for cancer immunotherapy 118. LCP mRNA vaccine encoding TRP2 were able to induce both antigen-specific cytotoxic T cell response and humoral immune response in a mouse model of melanoma. Besides, co-delivery of PD-L1 siRNA and mRNA vaccine resulted in the downregulation of PD-L1 in DCs, leading to the T cell activation and proliferation. Another preclinical report supported the combined immunotherapy of cancer vaccines and immune checkpoint blockades in non-immunogenic tumors 119. Lipid-coated calcium phosphate NPs containing CaP core, DOPA, DOTAP, and DSPE-PEG for delivering MUC1 mRNA with anti-CTLA-4 monoclonal antibody were designed to treat triple negative breast cancer. Additionally, researchers developed tetrasulfide-incorporated large-pore dendritic mesoporous organosilicon nanoparticles (DMONs) to address the challenge of mRNA delivery in hard-to-transfect cells 120. These nanoparticles were designed to exploit the intracellular glutathione (GSH) environment by consuming GSH, which efficiently enhanced mRNA delivery both *in vitro* and *in vivo*. By leveraging the unique properties of these nanoparticles, the delivery efficiency of mRNA was significantly improved in cells that are traditionally difficult to transfect. Nucleoside lipids for delivering mRNA have offered good compatibility and safety as mRNA can be loaded inside lipids through the hydrogen bonding interaction of base complementary pairings. PEGylated mRNA, obtained by hybridization with PEG-conjugated oligonucleotide (PEG-oligoRNA), was loaded with Lipofectamine LTX, leading to the structural stability *in vivo* 121. In a present study, a new class of nanocapsules, termed sugar-capsules composed of mannan (Mann-capsule) carrying mRNA were designed to elicit strong DC activation, mRNA translation, and antigen presentation (Figure 7) 122. This formulation boosted both CD4+ T and CD8+ T cell responses with antitumor efficacy *in vivo*, making it applicable for vaccines and immunotherapies utilizing pathogen-derived molecular patterns. Furthermore, biomimetic nanoparticles have also been explored for mRNA delivery. Phospholipid-derived nanoparticles, PL1, were found to be effectiveness in delivering costimulatory receptor mRNA (CD137 or OX40) to T cells (Figure 8) 123. Combining PL1-OX40 mRNA with anti-OX40 antibody resulted in a significantly enhanced antitumor activity compared to using anti-OX40 antibody alone in multiple tumor models. Currently, a PEG10 virus-like particle (VLP) platform was developed for efficient therapeutic delivery 124. By inserting genes of interest (the DNA template of mRNA) into the *Peg10* gene, this platform enabled potent gene editing by delivering gene-editing tools into cells. The selective endogenous encapsidation for cellular delivery (SEND) approach employed by the PEG10 VLP platform shows promise as an efficient therapeutic delivery modality.

## From preclinical studies to clinical trials

The primary objective of mRNA-based cancer vaccination is to induce or enhance an effective immune response against tumors 125, 126. This can be achieved through the delivery of synthetic mRNA encoding TAAs or TSAs using various approaches. Autologous dendritic cells can be genetically modified *ex vivo* with mRNA, or mRNA can be administered via formulated or non-formulated injections. Once vaccinated and taken up by APCs, mRNA is transported to the cytoplasm, undergoes antigen processing, and enters the MHC presentation pathway. Consequently, APCs present the antigens on MHC class I and II molecules, triggering the activation of CD8+ and CD4+ T cells, respectively. CD4+ T cells also stimulate antigen-specific B cells, leading to a humoral immune response. Since non-formulated, unprotected mRNA is susceptible to degradation by extracellular RNases, several pharmaceutical delivery systems have been developed to improve mRNA stability and facilitate its uptake by APCs. With ongoing research, well-optimized LNPs and polymers have been used for the targeted delivery of mRNAs to T cells for cancer immunotherapy 127, 128. For instance, conjugating antigen or CD4 antibodies to LNPs could realize the selective delivery of mRNAs to antigen-specific CD8+ T or CD4+ T cells, respectively 129, 130. These cell-specific mRNA delivery platforms allow for more precise and efficient therapies.

Among the mRNA delivery platforms, LNPs have gained clinical approval and demonstrate unique advantages, while other potential nanomaterial candidates are still emerging 16, 131. Different delivery materials have their own advantages and disadvantages, which should be considered based on specific needs 19, 132. In addition to the choice of delivery platform, the route of administration plays a crucial role in the effectiveness of mRNA vaccines. Generally, intramuscular (i.m.) and intradermal (i.d.) are widely utilized as the preferred routes of administration 133, 134. These routes offer distinct advantages, including the ability to elicit higher levels of immunity and longer-lasting effects compared to other administration methods. Intravenous (i.v.) administration, although less convenient due to liver first-pass metabolism, can be improved with enhanced delivery systems 135, 136. Ongoing efforts have led to various mRNA cancer vaccines using different vectors currently undergoing clinical trials (Table 1). The majority of mRNA vaccines that have undergone testing have been generally well-tolerated, although there have been occasional instances of localized reactions at the injection site. One noteworthy consideration regarding mRNA vaccines is the potential for systemic inflammation, which arises from their inherent capacity to act as immunostimulants by activating the TLR7/8 pathway and triggering type I IFN responses 4, 23. However, it is possible to limit the innate immune response to the specific injection site by implementing well-designed delivery systems and modifying the methods of administration.

The selection of appropriate antigens forms the foundation for the development of cancer vaccines 18. Typically, non-mutated shared tumor antigens are chosen as the primary targets for mRNA-based cancer vaccines. In multiple clinical trials, New York-ESO 1 (NY-ESO-1), Melanoma-associated antigen A3 (MAGE-A3), tyrosinase, and Trans-membrane phosphatase with tensin homology (TPTE) have been employed as TAAs for melanoma. For example, BNT111, a cancer vaccine based on the BioNTech FixVac platform, utilized a fixed combination of TAAs to elicit a potent and precise immune response against cancers 137. In a phase 1 clinical trial (NCT02410733) initiated in 2015, the intravenous administration of a tetravalent RNA-lipoplex (RNA-LPX) cancer vaccine, known as BNT111, was evaluated for its safety and tolerability in patients with advanced melanoma. The primary objective of this trial was to evaluate the vaccine's efficacy in targeting four TAAs in patients with advanced melanoma. The vaccine, referred to as Lipo-MERIT, was designed to elicit various immunological effects that were anticipated to contribute to its therapeutic impact. First, the RNA-LPX was home to APCs in lymphoid organs after i.v. injection, where they were rapidly taken up by professional APCs and the incorporated RNA was translated into antigenic proteins. These proteins were then processed and presented on both Human leukocyte antigen (HLA)-class I and HLA-class II molecules, triggering antigen-specific CD8+ and CD4+ T cell responses. Additionally, the Lipo-MERIT vaccine aimed to activate APCs through TLR signaling, leading to the induction of inflammatory cytokines and supporting the generation of tumor-antigen specific T cell responses. In 2021, the FixVac BNT111 vaccine was evaluated in an open-label, randomized phase 2 trial (NCT04526899) either as a single agent or in combination with cemiplimab in patients with anti-PD-1-refractory/relapsed, unresectable stage III or IV melanoma.

Furthermore, a phase 1/2 trial (NCT04382898) was started in 2019 to evaluate the safety, tolerability, immunogenicity, and preliminary efficacy of BNT112 cancer vaccine, which encoded five TAAs. The trial included patients with metastatic castration resistant prostate cancer (mCRPC) or high-risk, localized prostate cancer (LPC) and assessed the vaccine's efficacy alone or in combination with cemiplimab. In 2021, an open-label phase 2 randomized trial (NCT04534205) examined the efficacy of BNT113 in combination with pembrolizumab compared to pembrolizumab monotherapy as a first-line therapy for patients with unresectable recurrent or metastatic head and neck squamous cell carcinoma (HNSCC). This trial, known as AHEAD-MERIT, specifically targeted patients with HNSCC that were positive for human papillomavirus 16 (HPV16+) and expressed PD-L1. Another study conducted in 2019 (NCT04163094) was a phase 1 trial evaluating a liposome-formulated mRNA vaccine (BNT115) in combination with (neo-) adjuvant chemotherapy for ovarian cancer treatment. In 2022, a first-in-human (FIH) trial (NCT05142189) was conducted to assess the safety profile and determine the appropriate dosage of BNT116 as monotherapy and in combination with cemiplimab or docetaxel. The trial enrolled patients with advanced or metastasized non-small cell lung cancer (NSCLC). Subsequently, in 2023, a phase 2 study (NCT05557591) was initiated to evaluate the safety and efficacy of intravenous cemiplimab in combination with BNT116 compared to cemiplimab alone in patients with advanced NSCLC. The study specifically targeted NSCLC cases with tumors expressing PD-L1 ≥ 50%.

In addition to the FixVac vaccines, the mRNA lipoplex vaccine platform called individualized neoantigen-specific immunotherapy (iNeST) or BNT122 is being explored in several studies. iNeST includes mRNA lipoplex vaccines that encode individual tumor mutations, and the treatment (RO7198457) has been evaluated in clinical trials across multiple solid tumor diagnoses (NCT03289962, NCT03815058, NCT04486378, and NCT04161755) 18, 23. Also, iNeST was assessed in combination with another lipoplex-formulated mRNA encoding TAAs and RNA encoding p53 (NCT02316457) in patients with triple-negative breast cancer (TNBC). The mutanome engineered RNA immunotherapy (MERIT) study introduced a novel concept for individualized cancer immunotherapy (IVAC®) to treat each patient with specific and immunogenic RNA vaccines tailored to their tumors. This TNBC-MERIT trial implemented two complementary strategies, the WAREHOUSE and the IVAC® MUTANOME concept, resulting in two custom-made IVAC® investigational medicinal products (IMPs) for each patient. In 2020, there was a phase I/IIa, dose escalation trial (NCT04503278) with expansion cohorts to evaluate the safety and preliminary efficacy of CARvac, a CLDN6-encoding mRNA lipoplex vaccine, in patients with CLDN6-positive relapsed or refractory advanced solid tumors. CARVac was administered intravenously in combination with an autologous CLDN6 targeting CAR-T therapy, BNT211, with the aim of improving CAR-T therapy outcomes.

Neoantigens, which arise from somatic mutations in cancer cells, are typically specific to tumors and possess high immunogenicity, making them ideal targets for personalized vaccine development 138-140. As an example, in a study involving patients with high-risk cutaneous melanoma, two lipid nanoparticle mRNA cancer vaccines encoding multiple neoantigens (mRNA-4157) were evaluated. These vaccines aimed to harness the potential of neoantigens for immunotherapy in the context of melanoma treatment. In a phase 1 trial (NCT03313778), the safety, tolerability, and immunogenicity of mRNA-4157 alone were assessed in participants with resected solid tumors and in combination with pembrolizumab in participants with unresectable solid tumors. This treatment approach induced the generation of neoantigen-specific T cells and did not result in serious adverse events. Another phase 2 trial (NCT03897881) investigated whether postoperative adjuvant therapy with mRNA-4157 and pembrolizumab improved recurrence-free survival (RFS) compared to pembrolizumab alone in participants who had undergone complete resection of cutaneous melanoma and a high risk of recurrence. Additionally, a phase 1 clinical trial (NCT03948763) aimed to determine the safety and tolerability of mRNA-5671/V941 as a monotherapy and in combination with pembrolizumab in participants with Kirsten rat sarcoma viral oncogene (KRAS) mutant advanced or metastatic non-small cell lung cancer, colorectal cancer, or pancreatic adenocarcinoma. Two other studies involving mRNA-2416 (a lipid nanoparticle-encapsulated mRNA encoding Human OX40L; NCT03323398) and mRNA-2752 (a lipid nanoparticle-encapsulated mRNA encoding Human OX40L, IL-23, and IL-36γ; NCT03739931) explored intratumoral injection of these vaccines alone or in combination with immune checkpoint blockade for patients with solid tumor malignancies or lymphoma 141.

Protamine-formulated mRNA vaccines, known as RNActive® vaccines, have been extensively studied in several clinical trials 142, 143. These vaccines involved the use of nucleotide-modified mRNA molecules complexed with protamine, which enhanced both protein expression and immunogenicity. In 2012, a placebo-controlled phase 1/2 study (NCT01817738) examined an RNActive® vaccine called CV9104, which encoded six prostate cancer-specific antigens. The trial focused on patients with metastatic castration-resistant prostate cancer, aiming to assess the safety and efficacy of the vaccine. While the vaccine was found to be clinically safe, it did not demonstrate improvement in overall survival or progression-free survival compared to the placebo. Later in 2014, an open-label randomized phase 2 trial (NCT02140138) of RNActive® cancer vaccine (CV9104) was conducted in high risk and intermediate risk patients with prostate cancer. This study was the second clinical trial of the RNActive® vaccine, composed of six components encoding antigens overexpressed in prostate carcinoma compared to healthy tissue. Each of the six prostate-specific antigens encoded by CV9104 had the potential to induce adaptive immunity. Needle-free injection systems, like the Tropis® device for i.d. injection, have been employed to overcome the limitations associated with needle-based injections. Furthermore, CV9201, an mRNA-based vaccine for the treatment of human NSCLC, utilized CureVac's RNActive® technology (NCT00923312). CV9201 offered several advantages over alternative approaches, including high specificity, lack of restriction to the patient's MHC genotype, and the ability to exert activity without crossing the nuclear membrane. Then a phase 1b clinical trial evaluated an RNActive® vaccine treatment in combination with local irradiation in patients with stage IV NSCLC (NCT01915524) 144, 145. This study marked the introduction of the CV9202 lung cancer vaccine, composed of six RNActive® compounds, each encoding a different antigen overexpressed in NSCLC compared to healthy tissue. Lastly, in 2017, a phase 1/2 study (NCT03164772) was initiated to evaluate the safety and preliminary efficacy of the mRNA vaccine CV9202 plus one or two checkpoint inhibitors in subjects with NSCLC.

## Considerations and challenges for clinical translation

The field of mRNA vaccines is currently experiencing a surge in both basic and clinical research, as evidenced by a growing number of publications 146, 147. In recent years, preclinical and clinical studies have highlighted the efficacy of mRNA vaccines and their advantages over traditional vaccine platforms, including subunit, inactivated or live attenuated viruses, and DNA-based vaccines 148. While these studies have demonstrated the potency and versatility of mRNA vaccines in protecting against infectious pathogens or cancers, there are still challenges to overcome for their clinical application, which include good manufacturing practice production, stability, storage, and safety profiles 23, 28. Addressing these key points will contribute to the advancement and wider implementation of mRNA vaccines from bench to bedside.

To be specific, the manufacturing process is generally independent of the specific mRNA sequence, and it is primarily influenced by factors such as the RNA length, the nucleotide and capping chemistry, and the purification methods. However, certain sequence properties, particularly extreme length, can pose challenges in the manufacturing process 8, 17. After the mRNA is synthesized, multiple purification steps are employed to eliminate reaction components, including enzymes, free nucleotides, residual DNA, and truncated RNA fragments. In some mRNA platforms, the removal of double-stranded RNA (dsRNA) and other contaminants is crucial for ensuring the potency of the final products, as dsRNA can strongly induce interferon-dependent translation inhibition 23. Following purification, the mRNA is transferred to a final storage buffer and undergoes sterile filtration before being filled into vials for clinical use. RNA is susceptible to degradation through enzymatic and chemical pathways. Formulation buffers are carefully tested to ensure they are free from contaminating RNases and may contain additional components such as antioxidants and chelators, which minimize the impact of reactive oxygen species and divalent metal ions that cause mRNA instability.

The pharmaceutical formulation of mRNA is a rapidly evolving field of research and development 28, 149. While many products used in early-phase studies are typically stored at ultra-low temperatures (-70 °C), ongoing efforts are focused on developing formulations that remain stable at higher temperatures, making them more suitable for widespread vaccine distribution. For example, the RNActive® platform has demonstrated activity after lyophilization and storage at 5-25 °C for 3 years, and even at 40 °C for 6 months 143, 150. Stability of mRNA products can be improved by packaging within nanoparticles or by co-formulation with RNase inhibitors. Lipid-encapsulated mRNA has shown stability for at least 6 months, but long-term storage of such mRNA-lipid complexes in a non-frozen state requires further investigation 151.

Several mRNA vaccines have undergone extensive testing, ranging from phase I to IIb clinical studies, and have demonstrated a favorable safety profile, being generally safe and well tolerated 152, 153. However, in recent human trials, some mRNA platforms have shown moderate, and in rare instances, severe injection site or systemic reactions. Future preclinical and clinical studies will evaluate potential safety concerns, including local and systemic inflammation, biodistribution and persistence of expressed immunogens, potential toxicity of non-native nucleotides, and delivery vector components 105, 154. One particular concern is the potential for certain mRNA-based vaccines to trigger strong type I IFN responses, which are associated with inflammation and may potentially contribute to autoimmunity. Therefore, it is crucial to identify individuals who may be at a higher risk of autoimmune reactions prior to mRNA vaccination in order to take appropriate precautions. Continual evaluation of safety is necessary as different mRNA modalities and delivery systems are employed in human trials and tested on larger patient populations for the first time.

## Conclusions and future directions

mRNA vaccines have demonstrated their effectiveness in clinical settings for COVID-19 prevention. Inspired by this achievement, there is growing anticipation that additional mRNA-based vaccines and therapies will advance to the clinical translation phase. In this context, we offer an overview of delivery strategies for mRNA cancer vaccines. We delve into the technical hurdles associated with mRNA-based treatments, drawing connections to underlying biological mechanisms and their impact on preclinical and clinical outcomes. Furthermore, we underscore recent innovations and advancements in the pharmacology and delivery techniques of mRNA vaccines, which have the potential to accelerate the journey of mRNA-based cancer immunotherapy from laboratory research to practical clinical applications.

Recent advancements in comprehending and mitigating the innate immune response to mRNA have significantly expanded the applications of immunotherapy for cancer treatment. Conducting direct comparisons between different mRNA expression platforms will help determine the most suitable systems for both passive and active immunization. With a multitude of promising mRNA platforms available, conducting head-to-head comparisons would be immensely valuable in vaccine production, allowing researchers to allocate resources to those platforms that best align with each specific application. Additionally, further research is necessary to investigate how different animal species respond to mRNA vaccine components and inflammatory signals, as well as identifying the most effective immune signaling pathways in humans.

In conclusion, mRNA vaccination has demonstrated remarkable therapeutic potential in numerous preclinical and clinical trials 15, 155. Continued development of next-generation lipid nanoparticles and other delivery materials will further enhance the capabilities of mRNA-based therapies across a wide range of diseases, leading to improved healthcare worldwide. The availability of clinical data and resources from reputable companies and institutions is poised to propel valuable research in mRNA-based therapeutics, paving the way for a highly promising future for mRNA vaccines.

## Figures and Tables

**Table 1:** List of clinical trials of mRNA vaccines applying vectors for cancer therapy.

Vaccine type (Vector)	Name	mRNA encoding	Disease	Administration route	Start year (Status)	Phase	ClinicalTrials.gov identifier
Lipo-MERIT	BNT111	NY-ESO-1, MAGE-A3, tyrosinase, TPTE	Melanoma	i.v.	2015 (Active, not recruiting)	Phase I	NCT02410733
Lipo-MERIT	BNT111	NY-ESO-1, MAGE-A3, tyrosinase, TPTE	Unresectable stage III/IV melanoma	i.v.	2021 (Recruiting)	Phase II	NCT04526899
Lipo-MERIT	BNT112	5 PC TAAs	Prostate cancer (PC)	i.v.	2019 (Recruiting)	Phase I/II	NCT04382898
Lipo-MERIT	BNT113	Fixed combination of shared cancer antigens	Unresectable recurrent/metastatic head and neck squamous cell carcinoma (HNSCC)	i.v.	2021 (Recruiting)	Phase II	NCT04534205
Lipo-MERIT	BNT115	3 OC TAAs	Ovarian cancer (OC)	i.v.	2019 (Active, not recruiting)	Phase I	NCT04163094
Lipo-MERIT	BNT116	TAAs	Non-small cell lung cancer (NSCLC)	i.v.	2022 (Recruiting)	Phase I	NCT05142189
Lipo-MERIT	BNT116	TAAs	Advanced NSCLC	i.v.	2023 (Recruiting)	Phase II	NCT05557591
Lipo-MERIT	RO7198457	20 neoantigens	Melanoma, NSCLC, bladder/colorectal/renal cancers, breast cancer, head and neck cancer (HNC), other solid cancers	i.v.	2017 (Active, not recruiting)	Phase I	NCT03289962
Lipo-MERIT	RO7198457	20 neoantigens	Advanced melanoma	i.v.	2019 (Active, not recruiting)	Phase II	NCT03815058
Lipo-MERIT	RO7198457	20 neoantigens	Colorectal cancer stage II/III	i.v.	2021 (Recruiting)	Phase II	NCT04486378
Lipo-MERIT	RO7198457	20 neoantigens	Pancreatic cancer	-	2019 (Active, not recruiting)	Phase I	NCT04161755
Lipoplex	IVAC_W_bre1_uID, IVAC_W_bre1_uID/IVAC_M_uID	TAAs plus p53, 20 neoantigens	Triple-negative breast cancer (TNBC)	i.v.	2016 (Active, not recruiting)	Phase I	NCT02316457
Lipoplex	BNT211	CLDN6	Solid tumors	i.v.	2020 (Recruiting)	Phase I/II	NCT04503278
LNP	mRNA-2416	Human OX40L	Relapsed/Refractory solid tumors, ovarian cancer	i.t.	2017 (Terminated)	Phase I/II	NCT03323398
LNP	mRNA-2752	Human OX40L, IL-23, IL-36γ	Relapsed/Refractory solid tumor malignancies or lymphoma	i.t.	2018 (Recruiting)	Phase I	NCT03739931
LNP	mRNA-4157	~20 neoepitopes	Solid tumors	i.m.	2017 (Active, not recruiting)	Phase I	NCT03313778
LNP	mRNA-4157	~20 neoepitopes	Melanoma	i.v.	2019 (Recruiting)	Phase II	NCT03897881
LNP	V941	Kirsten rat sarcoma viral oncogene mutated proteins	Neoplasms, carcinoma, NSCLC, pancreatic neoplasms, colorectal neoplasms	i.m.	2019 (Completed)	Phase I	NCT03948763
LNP	NCI4650	Neoantigens	Melanoma, colon/ gastrointestinal/ genitourinary/ hepatocellular cancers	i.m.	2018 (Terminated)	Phase I/II	NCT03480152
Protamine	CV9104	Prostate-specific antigens	Prostate cancer	i.d.	2012 (Terminated)	Phase I/II	NCT01817738
Protamine	CV9104	Prostate-specific antigens	Prostate carcinoma	i.d.	2014 (Terminated)	Phase II	NCT02140138
Protamine	CV9201	MAGE-C1, MAGE-C2, NY-ESO-1, survivin, 5T4	NSCLC	i.d.	2009 (Completed)	Phase I/II	NCT00923312
Protamine	CV9202	6 different NSCLC associated antigens	NSCLC	i.d.	2013 (Terminated)	Phase I	NCT01915524
Protamine	CV9202	NY-ESO-1, MAGE-C1, MAGE-C2, 5T4, survivin, MUC1	NSCLC	i.d.	2017 (Completed)	Phase I/II	NCT03164772
